# miR-1306 induces cell apoptosis by targeting BMPR1B gene in the ovine granulosa cells

**DOI:** 10.3389/fgene.2022.989912

**Published:** 2022-09-23

**Authors:** Anwar Abdurahman, Wusimanjiang Aierken, Fei Zhang, Rahmantay Obulkasim, Jueken Aniwashi, Ablat Sulayman

**Affiliations:** ^1^ Shenzhen Key Laboratory of Marine Bioresources and Ecology, College of Life Sciences and Oceanography, Shenzhen University, Shenzhen, China; ^2^ College of Physics and Optoelectronic Engineering, Shenzhen University, Shenzhen, China; ^3^ College of Animal Science and Technology, Nanjing Agricultural University, Nanjing, China; ^4^ The Fifth Affiliate Hospital of Xinjiang Medical University, Urumqi, China; ^5^ Animal Diseases Control and Prevention Centre of Xinjiang Uygur Autonomous Region, Urumqi, China; ^6^ Ili Kazak Autonomous Prefecture Institute of Animal Science, Ili, China; ^7^ College of Animal Science and Technology, Xinjiang Agricultural University, Urumqi, China; ^8^ Institute of Animal Husbandry, Xinjiang Academy of Animal Science, Urumqi, China

**Keywords:** BMPR1B, granulosa cell, miR-1306, apoptosis, sheep

## Abstract

Bone morphogenetic protein receptor type-1B (BMPR1B) is one of the major gene for sheep prolificacy. However, few studies investigated its regulatory region. In this study, we reported that miR-1306 is a direct inhibitor of BMPR1B gene in the ovine granulosa cells (ovine GCs). We detected a miRNA response element of miR-1306 in the 3’ untranslated region of the ovine BMPR1B gene. Luciferase assay showed that the ovine BMPR1B gene is a direct target of miR-1306. qPCR and western blotting revealed that miR-1306 reduces the expression of BMPR1B mRNA and protein in the ovine granulosa cells. Furthermore, miR-1306 promoted cell apoptosis by suppressing BMPR1B expression in the ovine granulosa cells. Overall, our results suggest that miR-1306 is an epigenetic regulator of BMPR1B, and may serve as a potential target to improve the fecundity of sheep.

## Introduction

Bone morphogenetic protein (BMP)/Smad is one of the key signaling pathways for the regulation of mammalian fertility and plays important roles in gonadal development, steroid hormone production, follicular development, ovulation, luteal formation, and female reproductive diseases (Wang et al., 2010; Otsuka, 2018; Hoffmann et al., 2020; Wu et al., 2020). This signaling pathway is closely related to fecundity in sheep. Genes involved in this signaling pathway are widely expressed in ovarian tissue (Bahire et al., 2019), among which BMP15, and BMP receptor type-1B (BMPR1B) are the major genes of reproductive traits and their expression in ovarian tissues is related to fecundity (Xu et al., 2010; Foroughinia et al., 2017; Bahire et al., 2019).

In high and low fecundity Hu sheep ovarian antral follicles, the mRNA expression of BMPR1B, BMP4, BMPR2, GDF9, and Smad4 in high fecundity Hu sheep are significantly higher than that in low fecundity Hu sheep (Xu et al., 2010). Whole-genome sequencing and genome-wide association analysis in the pig show that a couple of members of BMP signaling pathways, like BMP5, BMP6, BMP7, BMP15, and BMPR1B are candidate genes related to reproductive traits (Schneider et al., 2014; Li et al., 2017). Among the major genes (BMP15, BMPR1B, GDF9, and B4GALT2) certified to affect sheep reproductive traits, only BMP15 and BMPR1B are core members of BMP signaling pathways (Abdoli et al., 2016).

BMPR1B was one of the most important major gene to be associated with prolificity in sheep, the A746G mutation (FecB) in the coding region is the only causal mutation site in BMPR1B (Mulsant et al., 2001). Ewes carrying FecB mutations have a higher ovulation rate and litter size (the set of offspring which mammals produce at one birth), fewer apoptotic granulocytes, and higher BMPR1B levels in the ovaries (Regan et al., 2015). The FecB gene has been detected in multiple breeds of sheep. It was found in the famous multiple-litter sheep breeds such as Hu sheep and Small-tailed Han sheep, among which the genotype frequencies of the three genotypes of Hu sheep FecB locus BB, B+ and ++ were 0.80, 0.16, and 0.04, respectively; The genotype frequencies of the three genotypes in Small Tail Han sheep were 0.26, 0.64 and 0.10, respectively (Montgomery et al., 2001; Tang et al., 2018). The FecB gene was also detected in Marwari, Bharat sheep (Kumar et al., 2006), Assaf sheep (Gootwine, 2011), and GMM sheep (Bahire et al., 2019). According to a recent study, BMPR1B is an important antiapoptotic factor in ovine GCs. BMPR1B can promote follicular development and increase fecundity via the inhibition of the apoptosis of Granulosa cells (Yao et al., 2019). Although the correlation between the FecB mutation polymorphism in the BMPR1B coding region and litter size has been shown, however few studies have investigated its regulatory region. In this study, we aimed to analyze the regulatory function of miRNAs on BMPR1B in the ovine granulosa cells to provide a basis for an investigation into the regulatory mechanism of BMPR1B transcription in sheep.

## Materials and methods

### Ethics statement

All experiments in this study were approved by the Animal Protection and Utilization Committee of Nanjing Agricultural University and were carried out in strict accordance with the policies of the National Laboratory Animal Administration (Order No. 2 of the China Science and Technology Commission, 14 November 1988, institution certification number: SYXK 2017-0027).

### Animals and samples collection

A total of 256 fresh sheep ovaries were collected from the Hauling slaughter house (Urumqi, Xinjiang). The ovaries were immediately soaked in 37°C saline that contained penicillin and streptomycin (Invitrogen, China) and taken to the laboratory for subsequent operations within 2 h.

### Cell culture

The isolation and culture of ovine Granulosa cells were carried out according to an experiment manual (Yao et al., 2019). After resuscitation, HEK293T cells were inoculated in Dulbecco’s modified Eagle’s medium that contained 1% double antibody (Invitrogen, China) and 10% fetal bovine serum (Invitrogen). Ovine follicular Granulosa cells and HEK293T cells were inoculated in a T25 cell culture flask (Corning) and cultured in a 37°C incubator that contained 5% CO_2_.

### Dual-luciferase assay

The cells were recaptured and placed in 6- and 12-well cell culture plates (Invitrogen, China) at the appropriate cell density. After 12 h, the cells were completely attached and transfected with Lipofectamine 3000 Transfection Reagent (Invitrogen, China), according to the kit instructions. The miR-1306-5p mimic sequence was CCA​CCU​CCC​CUG​CAA​ACG​UCC​A: mimics NC was UUC​UCC​GAA​CGU​GUC​ACG​UTT. Control or mimics were transfected one per well at a concentration of 20uM. Expression vector pcDNA3.1-BMPR1B was prepared previously by our group (Yao et al., 2019). Cells were transfected with 2 ug of pcDNA3.1 and pcDNA3.1-BMPR1B one per well. After 24 h of transfection, the cells were collected, and the fluorescence intensity of firefly and Renilla in each sample was measured by a dual-luciferase assay system kit (Promega company, US) and enzyme labeling instrument. The operation steps are detailed in the instructions.

### qRT-PCR

Total RNA was extracted from ovine Granulosa cells according to the instructions of a High Purity RNA Extraction Kit (Beijing Bioteke, China). The RNA thus obtained was reverse transcribed using PrimeScript RT Master Mix Reverse Transcriptase Kit (TaKaRa, Japan), and cDNA was generated using a SYBR Green Master Mix Kit (Vazyme, China) for quantitative analysis. The relative standard curve method (2^- △△t^ method) was used to calculate the relative expression of the target gene, based on the expression of the GAPDH internal reference gene.

### Western blotting

Antibodies for BMPR1B (ab155058) and GAPDH (ab9482) were obtained from Abcam (UK). Western blotting was performed according to a previously reported method (Liu et al., 2016).

### Apoptosis assay

The Annexin V-FITC/PI apoptosis detection kit (Vazyme, China) was used for granulosa cell apoptosis analysis. The experimental processes were operated following the kit instructions and the experimental process is detailed as follows. Cells were digested using trypsin and collected. Gently blow the cells with 500 μl PBS until they are resuspended and repeated once; after centrifugation of 1,000 g for 5 min, suck up the supernatant, resuspend the cells with 100 μl binding buffer, add 5 μl annexin V-FITC and 5 μl PI in turn in dark; After 10 min of incubation, add 400 μL binding buffer on the machine to measure the apoptosis rate of ovine Granulosa cells.

### Statistical analysis

Experimental data were analyzed for statistical significance with t-tests using SPSS v20.0 statistical software (SPSS Inc., Chicago, IL, United States). All experiments were performed in three biological replicates. Differences were considered significant and very significant at *p* < 0.05 and *p* < 0.01, respectively. Graphs were drawn using Prism 5.0 software.

## Results

### miR-1306 is a candidate miRNA that targets ovine BMPR1B

**FIGURE 1:**
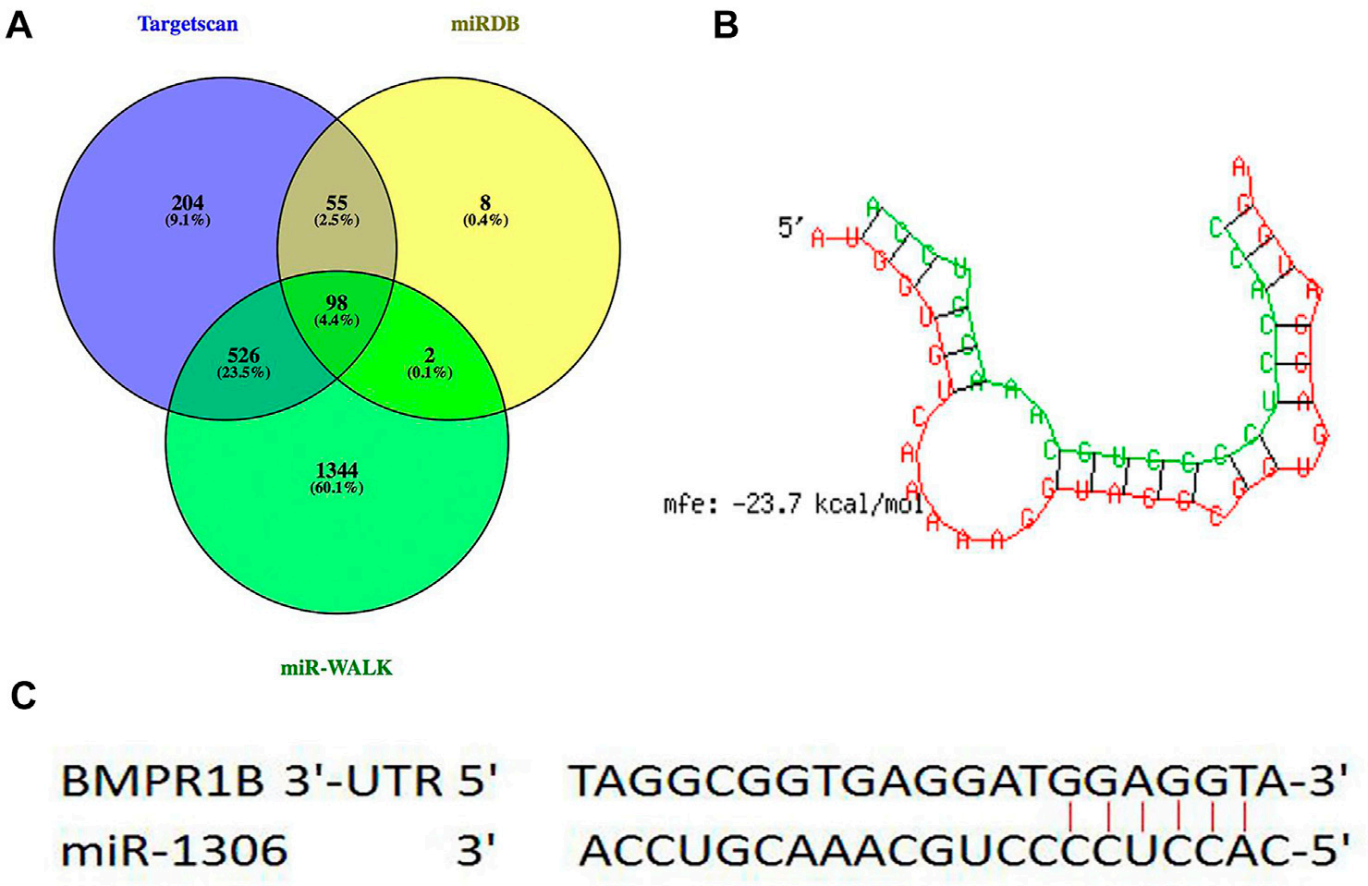
Candidate miRNAs that target BMPR1B. **(A)** miRNAs directly targeting BMPR1B were predicted using TargetScan, miRDB, and miR-WALK software. **(B)** RNAhybrid analysis. **(C)** Schematic diagram indicating the binding site sequences.

Through hybridization to incompletely complementary sequences in the 3' untranslated region (UTR) of their target messenger RNAs (mRNAs), miRNAs can negatively control gene transcription (Bartel, 2009; Djuranovic et al., 2012). Three types of online software (TargetScan, miRDB, and miR-walk) were used to predict the miRNAs in the 3'-UTR of BMPR1B, 98 common potential miRNAs were detected (Figure 1A). Among these, similar to the BMPR1B gene (Yao et al., 2019), miR-1306 has been identified as a key regulator of ovarian GC apoptosis in mammals (Yang et al., 2019). Results obtained using the online program RNAhybrid revealed that the binding site for miR-1306 is located at nucleotide 963–969 of the 3'-UTR of BMPR1B and that the minimum free energy for binding is -23.7 kcal/mol; this indicates that miR-1306 can stably bind with the BMPR1B 3′-UTR (Figures 1B,C). Based on these findings, miR-1306 was identified as a candidate miRNA targeting the ovine BMPR1B gene and was selected for further analysis.

### BMPR1B was a direct target of miR-1306 in ovine granulosa cells

**FIGURE 2:**
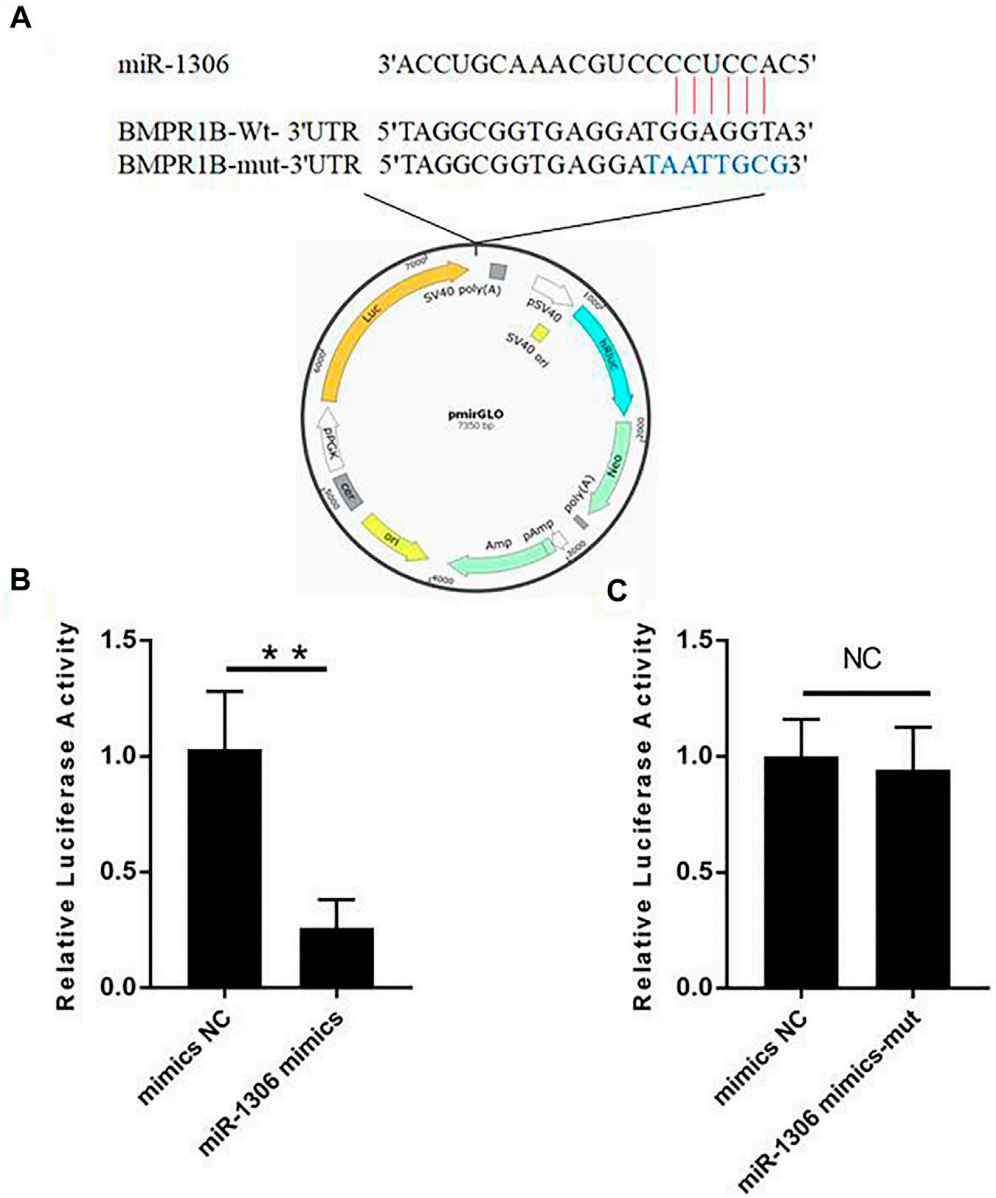
Hu sheep BMPR1B is a direct target of miR-1306. **(A)** BMPR1B 3'-UTR reporter vector. **(B, C)** Results of luciferase activity analyses. miR-1306 mimics and reporter vectors with wild-type **(B)** or mutant-type **(C)** miR-1306 binding sites were used to co-transfect HEK293T cells and determine luciferase activity. Bars represent the mean ± SEM of three repilicates.**p < 0.01. NS, no significant.

To investigate whether the BMPR1B gene of Hu sheep is targeted by miR-1306, we performed luciferase assays using wild-type and mutant-type BMPR1B gene 3'-UTR luciferase reporter vectors (Figure 2A). These vectors co-transfected with miR-1306 mimics. The assay results indicated that the luciferase activity in cells containing the wild-type vector was markedly reduced on co-transfection with miR-1306 mimics (Figure 2B), thereby indicating that miR-1306 can inhibit the transcriptional activity of the 3′-UTR region of BMPR1B. In contrast, we observed no significant changes when the miR-1306 mimics were co-transfected with the mutant-type vector (Figure 2C), thereby indicating that miR-1306 inhibits luciferase activity of Hu sheep GCs by binding directly to the 3'-UTR region of BMPR1B. Thus, our findings provide convincing evidence that the ovine BMPR1B gene is a direct target of miR-1306.

### miR-1306 regulated endogenous BMPR1B expression in ovine granulosa cells

**FIGURE 3:**
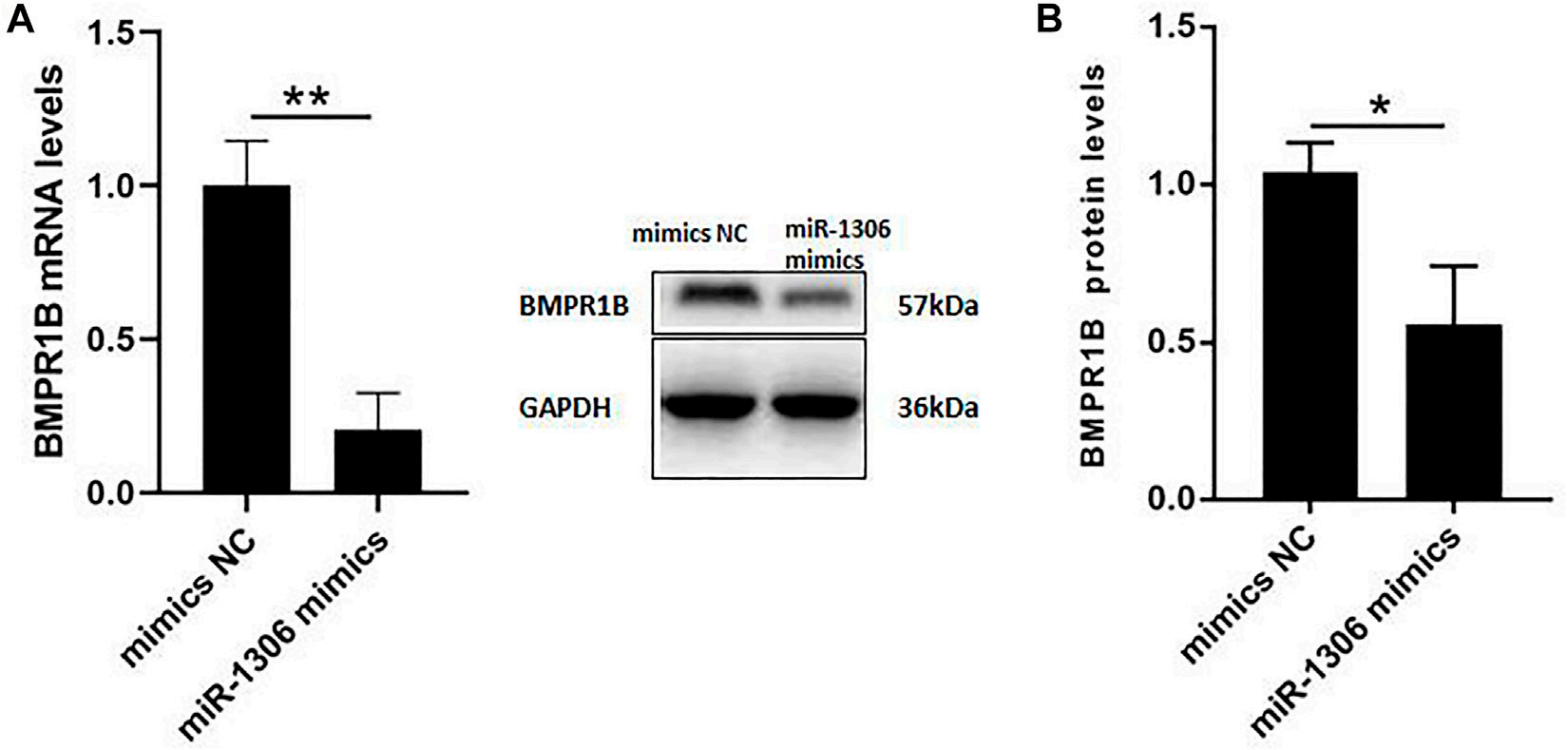
miR-1306 inhibits the expression of BMPR1B in sheep ovine Granulosa cells **(A)** BMPR1B mRNA levels. **(B)** BMPR1B protein levels. Bars represent the mean ± SEM of three repilicates. *p* < 0.05; **p < 0.01.

To further investigate the mechanisms whereby miR-1306 affects the expression levels of endogenous BMPR1B, ovine GCs were treated with miR-1306 mimics. As shown in Figure 3A, the overexpression of miR-1306 significantly down-regulated BMPR1B mRNA levels. Interestingly, in response to transfection with miR-1306 mimics, we observed that the protein levels of BMPR1B were markedly reduced in ovine GCs (Figure 3B), thereby indicating that miR-1306 can regulate BMPR1B expression in ovine GCs.

### miR-1306 promotes apoptosis in ovine granulosa cells

**FIGURE 4:**
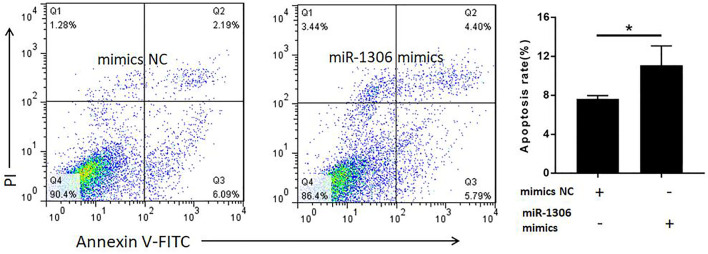
miR-1306 enhances apoptosis in ovine Granulosa cells miR-1306 promotes cell apoptosis. Bars represent the mean ± SEM of three repilicates. *p < 0.05.

To analyze the function of miR-1306 in ovine GCs, we treated these cells with miR-1306 mimics and mimics NC, respectively. FACS assays revealed that compared with the mimic NC group, overexpression of the miR-1306 mimic group markedly enhanced the apoptosis of ovine GCs (Figure 4), indicating that miR-1306 can function as an apoptotic factor in ovine GCs.

### miR-1306 promotes granulosa cells apoptosis by targeting BMPR1B

**FIGURE 5:**
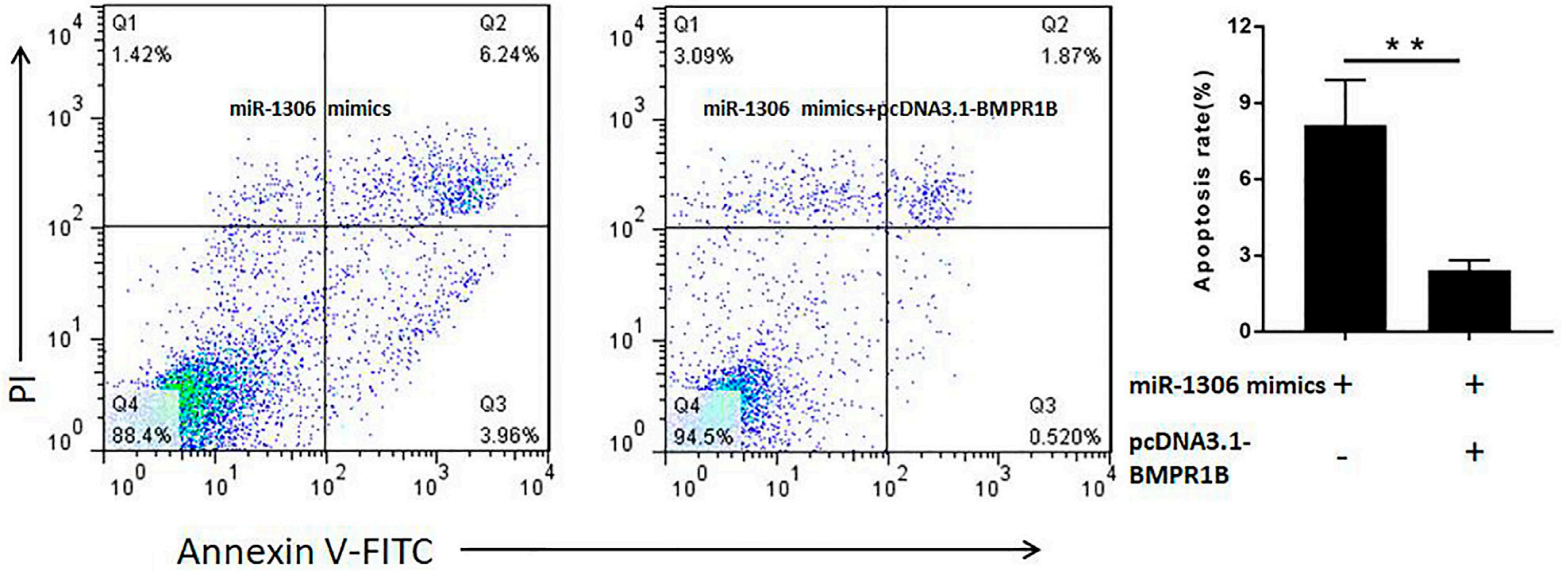
BMPR1B prevents the miR-1306-induced apoptosis of ovine Granulosa cells BMPR1B inhibits cell apoptosis caused by miR-1306. Bars represent the mean ± SEM of three repilicates.**p < 0.01.

To determine whether miR-1306 regulates ovine GC apoptosis via its interaction with BMPR1B, we co-transfected ovine GCs with miR-1306 mimics and the BMPR1B overexpression vector pcDNA3.1-BMPR1B *in vitro*. As shown in Figure 5, overexpression of BMPR1B can rescue the cell apoptosis caused by miR-1306 in the ovine GCs.

## Discussion

In the present study, we found that miR-1306 restrains the transcriptional activity of the BMPR1B by directly binding its 3′-UTR regions. Furthermore, the overexpression of miR-1306 significantly down-regulated BMPR1B mRNA and protein levels in ovine GCs. Finally, the apoptotic assay indicated that miR-1306 is an apoptotic factor in ovine granulosa cells, while the overexpression of BMPR1B prevents miR-1306-induced apoptosis.

3'-UTR is an important regulatory hub of mRNAs that can participate in the regulation of mRNAs level, translation, localization, stability, and polyadenylation status (Christine, 2018; Li F. et al., 2020). miRNA is a short non-coding RNA, that is, approximately 22 nt long and targets specific mRNAs 3'-untranslated regions (UTRs) and regulates gene expression (Bin Goh et al., 2012). miRNA can regulate various cellular processes, such as proliferation (Lozano-Velasco et al., 2015), differentiation (Wang et al., 2015), apoptosis (Cheng et al., 2005), and immune response (Okoye et al., 2014). Mechanistically, miRNAs mainly suppress the expression of target mRNAs via the reduction of translation or promotion of mRNA degradation (Du et al., 2020). In mammalian ovarian tissues, BMP/Smad is an important signaling pathway that affects reproductive performance and members of this pathway are direct targets for mRNAs. In ovine ovaries, BMPR2, SMAD1, SMAD4, and SMAD5 are potential target genes of miR-10b, miR-181a, miR-26a, miR-143, let-7a, miR-127, let-7f, and let-7c (Hu et al., 2016). The miR-17-92 cluster can regulate the cell proliferation and differentiation of bovine Granulosa cells by targeting BMPR2 (Andreas et al., 2016). miR-23a and miR-27a contribute to human Granulosa cell apoptosis by targeting SMAD5 (Nie et al., 2015). miR-130b targets SMAD5 to regulate the proliferation of Granulosa cells and cumulus cells, production of lactic acid in cumulus cells, biosynthesis of cholesterol, and ovulation in bovine ovaries (Sinha et al., 2017). SMAD4 is the most studied miRNA target gene among those in the ovarian BMP/Smad signaling pathway. miR-224 (Yao et al., 2010), miR-144 (Zhou et al., 2017), and miR-26b (Li et al., 2019) can regulate ovarian function by targeting SMAD4. In this study, we showed that miR-1306 direct targets BMPR1B gene in the ovine granulosa cells. However, few studies have investigated the regulation of BMPR1B by miRNAs. Currently, only studies that research how miR-125b targeting BMPR1B regulates apoptosis have been conducted in Granulosa cells (Yao et al., 2018; Yao et al., 2019). Together, our findings provide important insights into the epigenetic mechanisms underlying the regulation of BMPR1B expression in granulosa cells. miR-1306 is involved in various biological processes, for example, apoptosis, differentiation, proliferation, and pathological changes (Augustin et al., 2012; Du et al., 2018). The miR-1306 is differentially expressed in humans among those with Alzheimer’s disease, mild cognitive impairment, and vascular dementia, which suggests that miR-1306 plays an important role in the occurrence of these diseases (Li F. et al., 2020). Down-regulation of miR-1306 could aggravate the injury of OGD/rsh-sy5y *in vitro*; thus, miR-1306 plays a key role in cerebral I/R injury. Recently, a study found that Up-regulation of miR-1306 to lower cerebral ischemia/reperfusion injury *in vitro* by targeting BIK (Chen et al., 2019). Additionally, the expression of miR-1306 and congenital heart defects are closely related, which is significantly upregulated in twins (Pulakat et al., 2019). Chicken miR-1306, which generally targets Toll-interacting protein, has a key role in the host response against Salmonella enterica infection (Sun et al., 2019). Importantly, we predicted the binding site of miR-1306 on the 3'UTR of the BMPR1B gene. Further research found that the dual luciferase activity was significantly reduced after the binding site mutation of miR-1306 compared with the control group. These results suggest that miR-1306 directly binds to the 3'UTR of BMPR1B gene and reduces transcriptional activity. We observed that the mRNA and protein expression level of BMPR1B gene was strongly decreased after miR-1306 mimics was transfected into sheep ovarian granulosa cells. These data further demonstrated that miR-1306 binds to the 3'UTR of BMPR1B gene to regulate its expression.

Functionally, we showed that miR-1306 could promote granulosa cell apoptosis via directly binding to 3′UTR of BMPR1B gene. As is well known, the members of the BMP/Smad signaling pathway are associated with fecundity in domestic animals and are essential for steroidogenesis, follicular development, and ovulation and apoptosis (Abdoli et al., 2016; Liu et al., 2017; Li X. J. et al., 2020). Studies show that BMP2 (Liu et al., 2013), BMP4, BMP7 (Shimizu et al., 2012), and BMP15 (Shimizu et al., 2012) regulate granulosa cell apoptosis in mouse, cow, and human ovaries, respectively. BMPR2 and BMPR1B are two important receptors in the BMP/Smad signaling pathway. Previous studies have found that these two receptors have been found to regulate granulosa cell apoptosis in human (Cui et al., 2017) and sheep (Yao et al., 2019) ovaries, respectively.

In conclusion, our study provided evidence that miR-1306 regulates granulosa cell apoptosis through direct binding to the 3'UTR of the BMPR1B gene, and provides evidence of BMPR1B in regulating granulosa cell apoptosis in sheep.

## Conclusion

In summary, we showed that miR-1306 regulates the expression of BMPR1B in the ovine granulosa cells by directly binding to the 3'-UTR region. Moreover, we found that the BMPR1B gene is a functional target of miR-1306 and that miR-1306 induces ovine granulosa cell apoptosis. Our findings provide important insights into the molecular mechanisms underlying the regulation of BMPR1B expression and will provide a theoretical basis for improving the reproductive performance of sheep.

## Data Availability

The raw data supporting the conclusions of this article will be made available by the authors, without undue reservation.
